# Cinnamaldehyde Targets the LytTR DNA-Binding Domain of the Response Regulator AgrA to Attenuate Biofilm Formation of Listeria monocytogenes

**DOI:** 10.1128/spectrum.00300-23

**Published:** 2023-05-04

**Authors:** Xiaobing Jiang, Rui Kang, Tao Yu, Xiaojie Jiang, Hong Chen, Yiping Zhang, Yi Li, Hailei Wang

**Affiliations:** a Henan Engineering Laboratory for Bioconversion Technology of Functional Microbes, College of Life Sciences, Henan Normal University, Xinxiang, China; b School of Life Sciences & Basic Medicine, Xinxiang University, Xinxiang, China; c Key Laboratory of Biomedicine and Health Risk Warning of Xinxiang City, Xinxiang, China; Huazhong University of Science and Technology

**Keywords:** *Listeria monocytogenes*, cinnamaldehyde, AgrA, LytTR DNA binding domain, biofilm formation

## Abstract

The Agr quorum sensing (QS) system is known to contribute to biofilm formation in Listeria monocytogenes. Cinnamaldehyde, a natural food preservative, is considered an inhibitor of Agr-mediated QS in L. monocytogenes. However, the exact mechanism by which cinnamaldehyde acts on Agr remains unclear. In this study, we assessed the effects of cinnamaldehyde on the histidine kinase AgrC and the response regulator AgrA in the Agr system. AgrC kinase activity was not influenced by cinnamaldehyde, and binding between AgrC and cinnamaldehyde was not observed when microscale thermophoresis (MST) was performed, indicating that AgrC was not the target of cinnamaldehyde. AgrA is specifically bound to the *agr* promoter (P_2_) to activate the transcription of the Agr system. However, AgrA-P_2_ binding was prevented by cinnamaldehyde. The interaction between cinnamaldehyde and AgrA was further confirmed with MST. Two conserved amino acids, Asn-178 and Arg-179, located in the LytTR DNA-binding domain of AgrA, were identified as the key sites for cinnamaldehyde-AgrA binding by alanine mutagenesis and MST. Coincidentally, Asn-178 was also involved in the AgrA-P_2_ interaction. Taken together, these results suggest that cinnamaldehyde acts as a competitive inhibitor of AgrA in AgrA-P_2_ binding, which leads to suppressed transcription of the Agr system and reduced biofilm formation in L. monocytogenes.

**IMPORTANCE** Listeria monocytogenes can form biofilms on various food contact surfaces, posing a serious threat to food safety. Biofilm formation of L. monocytogenes is positively regulated by the Agr quorum sensing system. Thus, an alternative strategy for controlling L. monocytogenes biofilms is interfering with the Agr system. Cinnamaldehyde is considered an inhibitor of the L. monocytogenes Agr system; however, its exact mechanism of action is still unclear. Here, we found that AgrA (response regulator), rather than AgrC (histidine kinase), was the target of cinnamaldehyde. The conserved Asn-178 in the LytTR DNA-binding domain of AgrA was involved in cinnamaldehyde-AgrA and AgrA-P_2_ binding. Therefore, the occupation of Asn-178 by cinnamaldehyde suppressed transcription of the Agr system and reduced biofilm formation in L. monocytogenes. Our findings could provide a better understanding of the mechanism by which cinnamaldehyde inhibits L. monocytogenes biofilm formation.

## INTRODUCTION

Listeria monocytogenes is a Gram-positive bacterium and can cause listeriosis in humans and animals, with typical clinical manifestations including meningitis, septicemia, spontaneous abortion, and neonatal death (1). Listeriosis is a relatively rare but severe disease that is associated with high mortality rates (2). L. monocytogenes is distributed in a wide variety of environments and has been isolated from different types of foods, including eggs, meats, dairy products, and vegetables (3–5). Consumption of food contaminated by L. monocytogenes can cause sporadic cases or outbreaks of listeriosis (6, 7).

L. monocytogenes can adhere to food contact surfaces and subsequently form biofilms. Extracellular polymeric substances produced by L. monocytogenes in biofilms can protect bacterial cells from environmental threats, leading to the persistence of L. monocytogenes in food-processing environments (8). L. monocytogenes biofilms are potential sources of cross-contamination to food products, which seriously threatens food safety. Therefore, it is of great significance to control L. monocytogenes biofilms in the food industry.

In most bacteria, biofilm formation is associated with quorum sensing (QS) (9–11). QS is a bacterial cell-to-cell communication process that allows bacteria to behave coordinately by adjusting the expression of a specific set of genes (12). QS relies on the production, detection of, and response to the extracellular accumulation of signaling molecules called autoinducers (12). The QS system Agr/autoinducing peptide (AIP) has been identified in L. monocytogenes (13). Some scholars believe that the Agr/AIP system of L. monocytogenes, strictly speaking, is not dedicated to QS (14), because experimental evidence indicates that the Agr/AIP system is not a mechanism to assess cell density to make a coordinated response of the whole population in L. monocytogenes (15). Although the discussion about whether Agr/AIP in L. monocytogenes is dedicated to QS is controversial, we refer to it as a QS system for the sake of description in this study. Agr/AIP is composed of the four-gene operon *agrBDCA*, and its expression is controlled by the P_2_ promoter upstream from *agrB* (13). The four genes, *agrB*, *agrD*, *agrC*, and *agrA*, encode the precursor-processing enzyme AgrB, precursor peptide AgrD, histidine kinase AgrC, and response regulator AgrA, respectively (13). Among the four proteins, AgrC and AgrA constitute a two-component signal transduction system (TCS). The precursor peptide AgrD of L. monocytogenes is processed by AgrB, and the mature AIP is released outside the cells. When the AIP reaches a certain concentration threshold, it specifically binds to the receptor AgrC, which activates AgrC self-phosphorylation (14). Then, the phosphate group is transferred to AgrA, and phosphorylated AgrA can regulate the downstream target genes by binding to the promoter DNA (14). The Agr system positively regulates L. monocytogenes biofilm formation (15, 16). Thus, interfering with the Agr system provides an alternative strategy for controlling L. monocytogenes biofilms and improving food safety.

Cinnamaldehyde, a phenylpropanoid compound, is the major bioactive component of Chinese cinnamon (17). Due to its good antibacterial activity and safety profile, cinnamaldehyde has been approved as a food preservative in China. Many previous studies have reported that cinnamaldehyde is an inhibitor of AI-2-based QS (18–20) and can also inhibit QS-controlled phenotypes, including biofilm formation and virulence in Vibrio spp. (20), Pseudomonas fluorescens (17), Escherichia coli (21), and Staphylococcus aureus (22). Recently, Liu et al. (23) found that biofilm formation and the expression levels of the *agr* genes in L. monocytogenes were reduced after the addition of cinnamaldehyde, suggesting that cinnamaldehyde interferes with the L. monocytogenes Agr system. However, its exact mechanism of action remains unclear.

To explore the molecular mechanism by which cinnamaldehyde inhibits the Agr system in L. monocytogenes, we investigated the effects of cinnamaldehyde on AgrC and AgrA in this study. We conclude that AgrA, rather than AgrC, is the target of cinnamaldehyde. The conserved Asn-178 of AgrA was identified as the key amino acid site in cinnamaldehyde-AgrA and AgrA-P_2_ binding. Our findings suggest that cinnamaldehyde can prevent AgrA from binding to the P_2_ promoter of the Agr system. This study provides novel insights into the inhibition of L. monocytogenes biofilm formation by cinnamaldehyde.

## RESULTS

### Cinnamaldehyde is an inhibitor of the Agr QS system.

**FIG 1:**
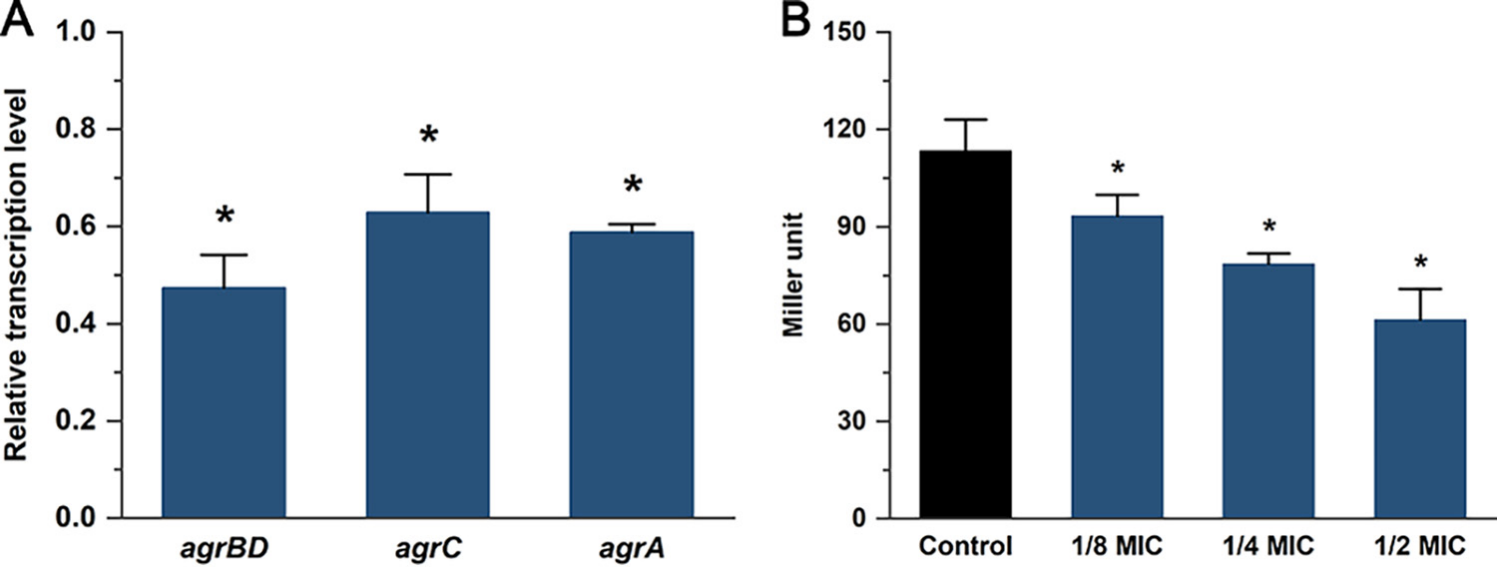
Cinnamaldehyde is an inhibitor of the Agr QS system in L. monocytogenes EGD-e. (A) Relative transcription levels of *agr* genes in the presence of cinnamaldehyde (1/2 MIC) were determined by qRT-PCR. The results are presented as fold changes relative to the transcription level of the target gene in L. monocytogenes EGD-e without cinnamaldehyde. (B) The effect of cinnamaldehyde at different concentrations (1/8 MIC, 1/4 MIC, and 1/2 MIC) on *agr* promoter (P_2_) activity was evaluated using the β-galactosidase assay. Error bars represent the standard deviations of triplicate experiments (*n *= 3). An asterisk indicates a value statistically different from that of the control at *P < *0.05.

The MIC of cinnamaldehyde against L. monocytogenes strain EGD-e was 500 μg/mL. The relative transcription levels of *agrBDCA* in EGD-e exposed to 1/2 MIC of cinnamaldehyde were measured by using quantitative real-time PCR (qRT-PCR). As shown by the results in Fig. 1A, the transcription levels of *agrBD*, *agrC*, and *agrA* were significantly decreased (*P < *0.05) in the presence of cinnamaldehyde. Additionally, the effects of cinnamaldehyde on the activity of the *agr* promoter P_2_ were investigated. Cinnamaldehyde at 1/8 MIC, 1/4 MIC, and 1/2 MIC reduced P_2_ activity by 17.6, 30.7, and 45.9%, respectively (Fig. 1B). These data suggest that the transcription of the *agr* operon is suppressed by cinnamaldehyde.

### Cinnamaldehyde has no effect on the kinase activity of AgrC.

**FIG 2:**
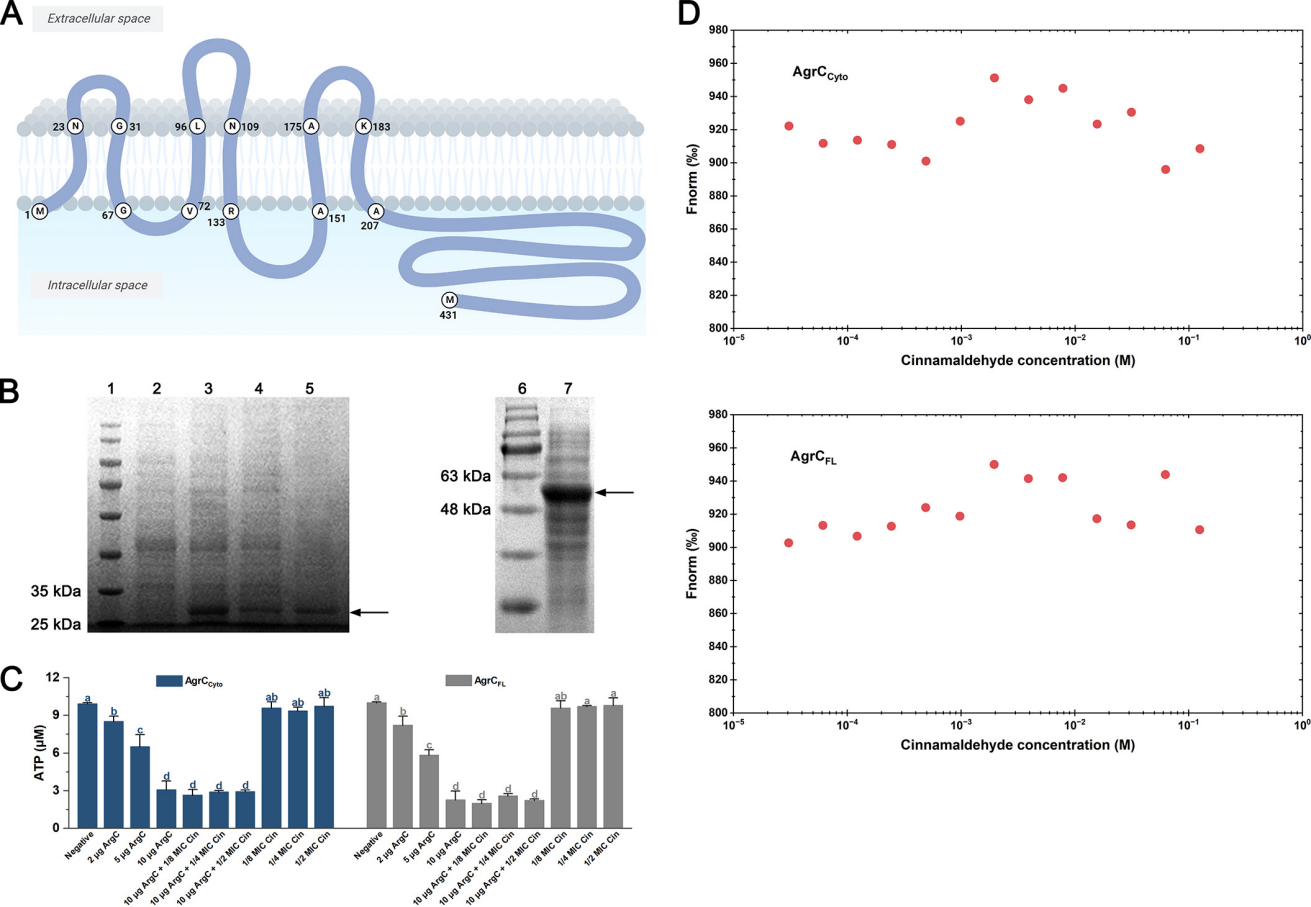
Cinnamaldehyde does not affect the kinase activity of AgrC, nor does it bind to AgrC *in vitro*. (A) The schematic shows the proposed transmembrane topology of AgrC. (B) SDS-PAGE analysis of recombinant proteins of AgrC_Cyto_ and AgrC_FL_. Lanes 1 and 6, molecular mass markers; lane 2, expression of recombinant AgrC_Cyto_ in E. coli without IPTG; lanes 3 and 4, expression of recombinant AgrC_Cyto_ in E. coli induced by IPTG; lane 5, expression of purified AgrC_Cyto_; lane 7, expression of purified AgrC_FL_. Arrows indicate the proteins of interest. (C) Effect of cinnamaldehyde at different concentrations (1/8 MIC, 1/4 MIC, and 1/2 MIC) on kinase activity of AgrC_Cyto_ and AgrC_FL_. Negative, 10 μM ATP was added to the reaction system and the kinase reaction was conducted in the presence of AgrC. Cin, cinnamaldehyde. Different letters on top of the bars represent significant differences (*P < *0.05). Error bars represent the standard deviations of triplicate experiments (*n *= 3). (D) Determination of interactions between cinnamaldehyde and AgrC_Cyto_ or AgrC_FL_ by MST. Fnorm, normalized fluorescence.

Since AgrC in L. monocytogenes is a transmembrane receptor, the determination of its membrane topology model is an important first step in understanding its structure-function relationships. In this study, we analyzed the transmembrane segments (TMSs) of AgrC using the TMHMM-2.0 program (https://services.healthtech.dtu.dk/service.php?TMHMM-2.0). AgrC of L. monocytogenes contains two distinct domains: an N-terminal sensor module (residues 1 to 206) and a C-terminal cytoplasmic histidine kinase domain (residues 207 to 431). According to the prediction results of TMHMM, the sensor module comprises six TMSs: residues 2 to 22 (in-to-out), residues 32 to 66 (out-to-in), residues 73 to 95 (in-to-out), residues 110 to 132 (out-to-in), residues 152 to 174 (in-to-out), and residues 184 to 206 (out-to-in) (Fig. 2A). AgrC contained three short extracellular loops with lengths of 9, 14, and 9 amino acids (Fig. 2A). The histidine kinase domain of AgrC (residue 207 to the end) was located in the periplasm as an intracellularly exposed tail.

To investigate whether AgrC was the target of cinnamaldehyde, AgrC_Cyto_ (the partial protein with the cytoplasmic domain) and AgrC_FL_ (the full-length protein of AgrC) were expressed in the present study. Purified AgrC_Cyto_ and AgrC_FL_ were loaded onto SDS-PAGE gels for analysis, and strong bands with molecular masses of approximately 25 and 48 kDa, respectively, were observed (Fig. 2B). AgrC_Cyto_ (residues 207 to 431) has two subdomains, a membrane-proximal dimerization and histidine phosphotransfer (DHp) subdomain and the catalytic and ATP binding (CA) subdomain, which can bind and hydrolyze ATP and autophosphorylate. The kinase activity of AgrC_Cyto_ was assayed in our study, and it was inversely related to the residual amount of ATP. In the reaction system, the initial concentration of ATP was 10 μM. After incubation, the remaining ATP was reduced with increasing concentrations of AgrC_Cyto_ (Fig. 2C), suggesting that the purified AgrC_Cyto_ in this study exhibited *in vitro* kinase activity, as expected. The addition of cinnamaldehyde at different concentrations did not affect the residual amount of ATP in the reaction system (Fig. 2C), indicating that cinnamaldehyde had no effect on the kinase activity of AgrC_Cyto_ *in vitro*. AgrC_FL_ was also subjected to the kinase assay, and similar results were observed (Fig. 2C).

### Cinnamaldehyde does not bind to AgrC *in vitro*.

Microscale thermophoresis (MST) was performed to confirm the interaction between cinnamaldehyde and AgrC. As shown by the results in Fig. 2D, an S-shaped binding curve was not observed based on the MST data of cinnamaldehyde and AgrC_Cyto_, suggesting that cinnamaldehyde did not bind to AgrC_Cyto_. Next, the interaction between cinnamaldehyde and AgrC_FL_ was evaluated by MST. Again, no binding was observed between them (Fig. 2D). Therefore, our results suggest that AgrC is not the target of cinnamaldehyde.

### Cinnamaldehyde interferes with the interaction between AgrA and P_2_ by competitive binding to AgrA.

**FIG 3:**
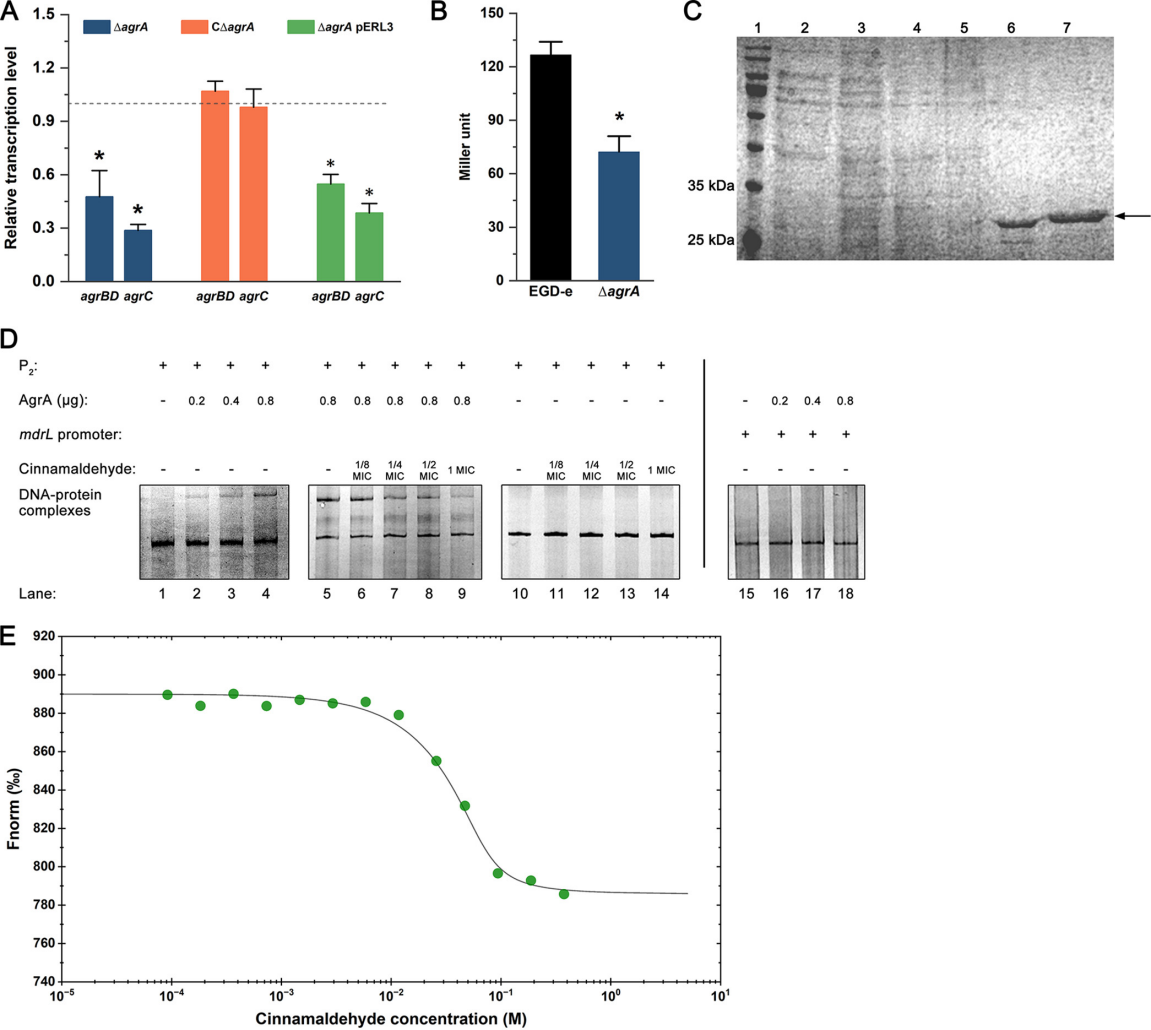
Cinnamaldehyde is a competitive inhibitor of AgrA-P_2_ binding. (A) Relative transcription levels of *agrBD* and *agrC* in the Δ*agrA* mutant compared to L. monocytogenes wild-type strain EGD-e. The results are presented as fold changes relative to the transcription level of the target gene in EGD-e. (B) P_2_ promoter activity in EGD-e and the Δ*agrA* mutant. (C) SDS-PAGE analysis of AgrA from recombinant E. coli strains and purified proteins. Lane 1, molecular mass markers; lane 2, expression of recombinant AgrA in E. coli without IPTG; lanes 3 to 5, expression of recombinant AgrA in E. coli induced by IPTG; lanes 6 and 7, expression of purified AgrA. The arrow indicates the protein of interest. (D) AgrA affinity for P_2_ promoter is disrupted by cinnamaldehyde. The indicated amount of purified AgrA protein was incubated with P_2_ promoter DNA (lanes 1 to 4). Cinnamaldehyde was added to the binding reaction mixture (lanes 5 to 9) to disrupt the AgrA-P_2_ interaction. The addition of cinnamaldehyde to the binding reaction mixture without AgrA (lanes 10 to 14) was used to detect an interaction between cinnamaldehyde and P_2_. An unrelated DNA sequence (*mdrL* promoter) was used as a negative control (lanes 15 to 18). (E) Quantification of the binding affinity between cinnamaldehyde and AgrA by MST. Error bars represent the standard deviations of triplicate experiments (*n *= 3). An asterisk indicates a value statistically different from that of the wild-type strain, EGD-e (*P < *0.05).

To investigate the effect of *agrA* mutation on *agr* operon transcription, an Δ*agrA* gene deletion mutant was constructed in this study. As shown by the results in Fig. 3A, the relative transcription levels of *agrBD* and *agrC* in the Δ*agrA* mutant were significantly lower (*P < *0.05) than those of the wild-type strain EGD-e, and complementation of the *agrA* mutation restored the transcription of *agrBD* and *agrC*. In addition, the activity of the *agr* promoter P_2_ in the Δ*agrA* mutant was clearly decreased (*P < *0.05) (Fig. 3B). These data suggest the positive regulation of the Agr system by AgrA.

Subsequently, the AgrA protein was expressed and purified (Fig. 3C), and an electrophoretic mobility shift assay (EMSA) was performed to investigate the interaction between purified AgrA and the P_2_ promoter. Shifted bands of protein-DNA complexes were observed in the gel, indicating binding of the recombinant AgrA protein to the P_2_ promoter (Fig. 3D). However, binding of AgrA to an unrelated DNA sequence of the same GC content was not observed, indicating that the binding of the recombinant AgrA protein to the P_2_ promoter was specific. These results suggest that AgrA binds to the P_2_ promoter to positively regulate the Agr system.

To investigate the effect of cinnamaldehyde on binding between AgrA and the P_2_ promoter, cinnamaldehyde at different concentrations was added to the binding reaction mixture. As shown in Fig. 3D, the shifted bands of AgrA-P_2_ complexes became weaker with increasing cinnamaldehyde concentrations. Furthermore, our results showed that ethanol, which was the solvent in the cinnamaldehyde solution, did not affect the interaction between AgrA and P_2_ (Fig. 3D). Additionally, binding between cinnamaldehyde and P_2_ was not observed (Fig. 3D). These results suggest that cinnamaldehyde disrupts the interaction between AgrA and P_2_.

MST showed that cinnamaldehyde bound to L. monocytogenes AgrA with a dissociation constant (*K_d_*) of 55.93 ± 2.38 (mean ± standard deviation) (Fig. 3E), suggesting that cinnamaldehyde is the competitive inhibitor of AgrA-P_2_ binding.

### Asn-178 and Arg-179 in the LytTR domain of AgrA are critical for cinnamaldehyde-AgrA binding.

**FIG 4:**
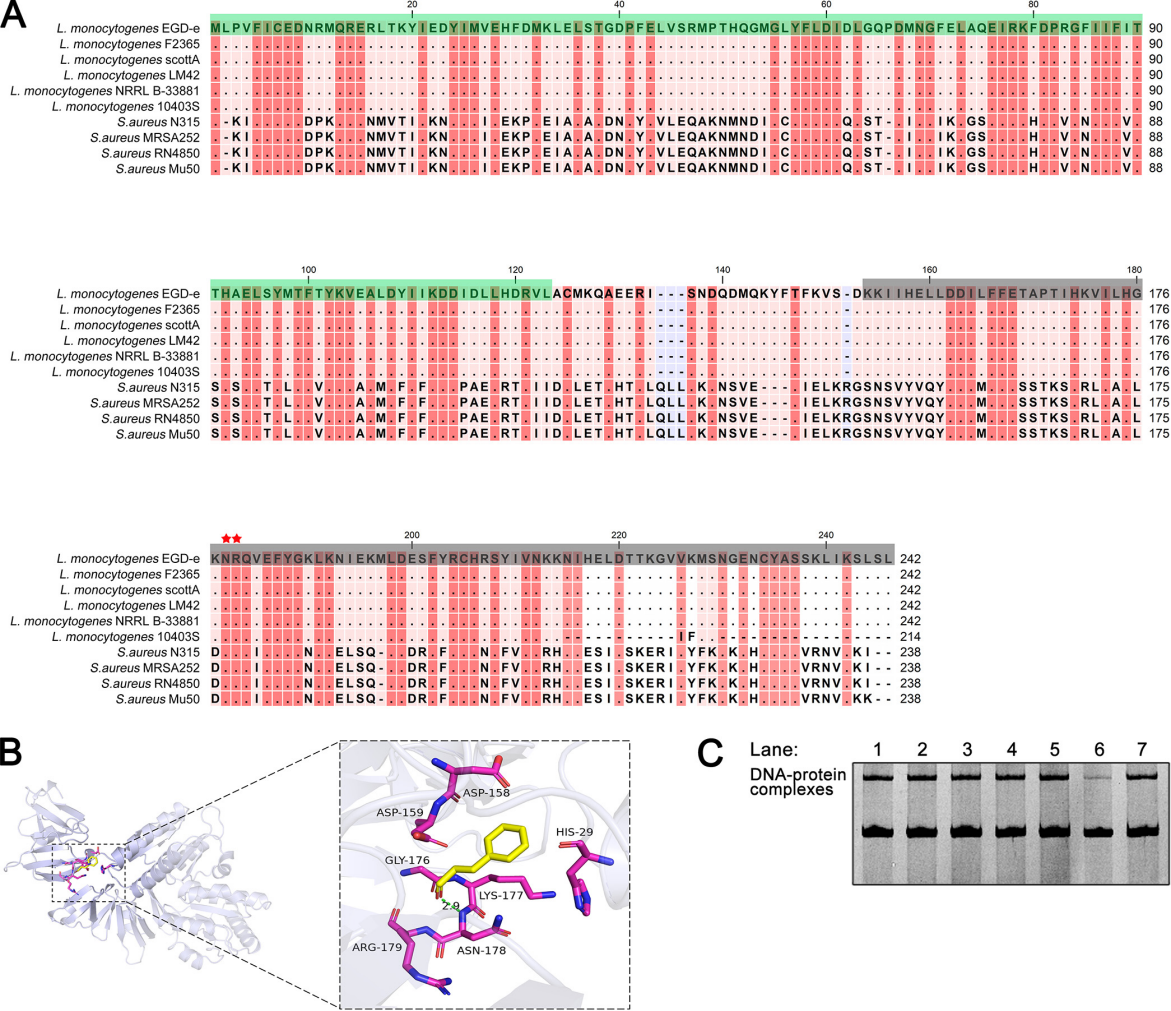
(A) Sequence alignment of AgrA proteins. The N-terminal phosphoacceptor receiver domain (residues 1 to 123) and C-terminal LytTR DNA-binding domain (residues 150 to 242) are highlighted in green and gray, respectively. The key sites Asn-178 and Arg-179 are indicated with red five-pointed stars. (B) Molecular docking of cinnamaldehyde with AgrA. (C) Interaction between mutated AgrA and P_2_ promoter by EMSA. One microgram of protein was incubated with 200 ng of DNA in a binding buffer. Lane 1, AgrA; lane 2, AgrA_D158A_; lane 3, AgrA_D159A_; lane 4, AgrA_G176A_; lane 5, AgrA_K177A_; lane 6, AgrA_N178A_; lane 7, AgrA_R179A_.

AgrA in L. monocytogenes EGD-e comprises 242 amino acids and belongs to the LytTR family of response regulators. The AgrA of L. monocytogenes EGD-e shares 42% identity with the AgrA of S. aureus N315 (Fig. 4A). Based on the amino acid sequence analysis, AgrA contains an N-terminal phosphoacceptor receiver domain (residues 1 to 123) and a C-terminal LytTR DNA-binding domain (residues 150 to 242) (Fig. 4A).

Previous studies have reported that AgrA dimerizes and binds to DNA (24, 25). Thus, AgrA in homodimeric form was used for docking analysis of the interaction between cinnamaldehyde and AgrA. As shown in Fig. 4B, stabilization of the complex was mediated by a hydrogen bond and hydrophobic forces. The binding energy between cinnamaldehyde and AgrA was −4.7 kcal/mol. Cinnamaldehyde formed a hydrogen bond to Asn-178 with a 2.9-Å distance and hydrophobic interactions with His-29, Asp158, Asp159, Gly-176, Lys-177, and Arg-179 of L. monocytogenes AgrA. All predicted binding sites, except for His-29, were located in the DNA-binding domain of AgrA.

To confirm the role of these sites in the cinnamaldehyde-AgrA interaction, the seven amino acids were mutated to alanine, and the binding ability of the mutant protein to cinnamaldehyde was detected by MST. As shown by the results in Table 1, AgrA_H29A_ (AgrA bearing the mutation of His to Ala at position 29), AgrA_D158A_, AgrA_D159A_, AgrA_G176A_, and AgrA_K177A_ showed binding abilities to cinnamaldehyde similar to that of wild-type AgrA. Compared with that of cinnamaldehyde-AgrA, higher *K_d_* values were observed in cinnamaldehyde-AgrA_N178A_ and cinnamaldehyde-AgrA_R179A_, suggesting lower binding abilities of AgrA_N178A_ and AgrA_R179A_ to cinnamaldehyde (Table 1). Additionally, Asn-178 and Arg-179 are highly conserved among L. monocytogenes strains (Fig. 4A), indicating that they are key sites for the interaction between cinnamaldehyde and AgrA in L. monocytogenes.

**TABLE 1:** *K_d_* values for cinnamaldehyde binding to AgrA determined by MST

Protein	Mean *K_d_* ± SD (μM)
AgrA	55.93 ± 2.38
AgrA_H29A_	57.70 ± 6.47
AgrA_D158A_	61.97 ± 4.80
AgrA_D159A_	59.02 ± 3.44
AgrA_G176A_	53.18 ± 1.52
AgrA_K177A_	58.32 ± 4.42
AgrA_N178A_	138.4 ± 11.4
AgrA_R179A_	174.6 ± 17.8

### Asn-178 is essential for AgrA-P_2_ binding.

We further investigated whether the mutation of the six predicted sites affected the binding between AgrA and P_2_. Compared with AgrA-P_2_, only the AgrA_N178A_-P_2_ complex showed a weaker shifted band, suggesting an important role of Asn-178 in AgrA-P_2_ binding (Fig. 4C). These findings provide strong evidence that Asn-178 of AgrA is critical for cinnamaldehyde-AgrA binding and AgrA-P_2_ binding.

### Cinnamaldehyde inhibits L. monocytogenes biofilm formation.

**FIG 5:**
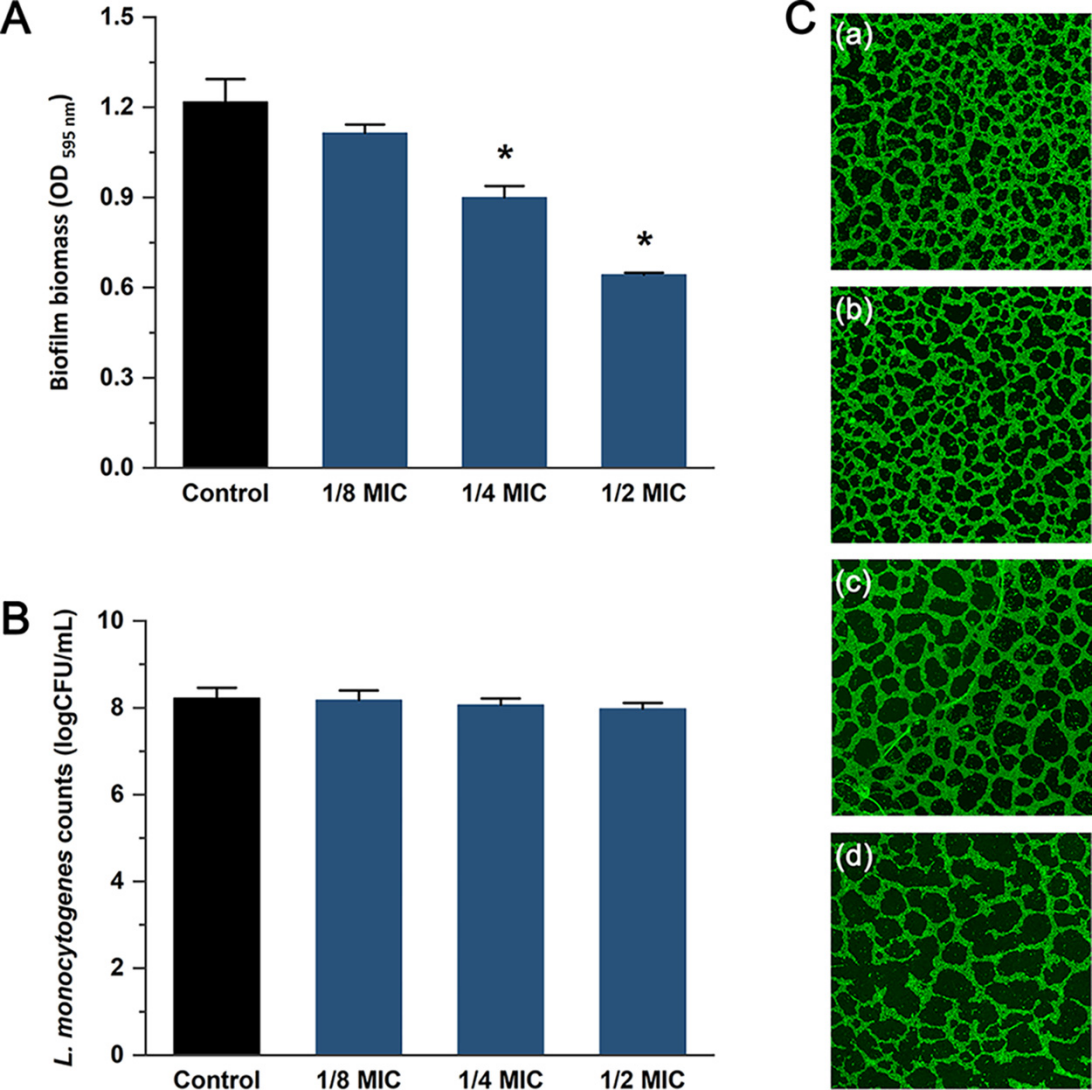
Cinnamaldehyde inhibits biofilm formation of L. monocytogenes EGD-e. (A) Biofilm biomass measured by the crystal violet staining method. (B) The number of planktonic cells in the culture supernatant. (C) CLSM images of the biofilms. (a) Untreated control; (b to d) concentrations of cinnamaldehyde were 1/8 MIC, 1/4 MIC, and 1/2 MIC. Error bars represent the standard deviations. Control refers to biofilms incubated in BHI broth without cinnamaldehyde. An asterisk indicates a value statistically different from that of the control (*P < *0.05).

Biofilms of L. monocytogenes EGD-e were quantified using the crystal violet staining method. As shown by the results in Fig. 5A, only a slight reduction (8.6%; *P > *0.05) in biofilm biomass was observed when the strain was exposed to 1/8 MIC of cinnamaldehyde, while cinnamaldehyde at concentrations of 1/4 MIC and 1/2 MIC obviously reduced (*P < *0.05) biofilm biomass, by 26.1 and 47.2%, respectively. Compared with the control (EGD-e grown in brain heart infusion [BHI] medium without cinnamaldehyde), the addition of cinnamaldehyde did not affect the viability of the planktonic cells (Fig. 5B). Biofilm morphology was also observed by using confocal laser scanning microscopy (CLSM) in this study. CLSM images demonstrated that L. monocytogenes biofilms incubated without cinnamaldehyde were complete and dense. However, decreased adherence and increased biofilm dispersal were observed in the presence of cinnamaldehyde (Fig. 5C).

## DISCUSSION

The Agr system is present across firmicute species, and it has been well studied in S. aureus (26). To date, many inhibitors targeting the Agr system of S. aureus have been reported and the action modes of several inhibitors have been clarified (27–32). However, data on inhibitors of the Agr system of L. monocytogenes are scarce. In this study, both the transcription levels of the *agr* genes and the activity of the *agr* promoter P_2_ in L. monocytogenes were inhibited by a sub-MIC amount of cinnamaldehyde. Liu et al. (23) have reported similar results, showing the suppressed expression of the *agr* genes in the presence of cinnamaldehyde at 1/4 MIC. These findings suggest that cinnamaldehyde can inhibit biofilm formation of L. monocytogenes by acting as an inhibitor of the Agr QS system. However, the exact mechanism by which cinnamaldehyde acts on Agr remains unclear.

AgrC acts as a sensor for the recognition of environmental signals and the transduction of signals into the cytoplasm (33, 34). Therefore, AgrC is considered a compelling target for the development of novel inhibitors against the Agr QS system (27). Different inhibitors targeting AgrC in S. aureus have been synthesized to interfere with the AgrC/AIP interaction and attenuate staphylococcal virulence, such as cyclic peptide mimetics of AIP (28, 35), AIP-sequestering antibodies (36, 37), and nonpeptidic small molecules (29). In L. monocytogenes, AgrC, composed of 431 amino acids, contains a sensor domain that can recognize AIP and a cytoplasmic histidine kinase domain that has the function of transferring the phosphate group of ATP to the histidine residue located in the DHp subdomain (38). If cinnamaldehyde binds to the cytoplasmic histidine kinase domain of AgrC (AgrC_Cyto_), self-phosphorylation of AgrC_Cyto_ can be inhibited. However, our results showed that cinnamaldehyde had no effect on the phosphorylation level of AgrC_Cyto_. Furthermore, the results from MST provided direct evidence that cinnamaldehyde did not bind to AgrC_Cyto_. To determine whether cinnamaldehyde binds to the sensor domain of AgrC, the full-length protein, AgrC_FL_, was expressed and purified. However, binding between cinnamaldehyde and AgrC_FL_ was not observed in this study, suggesting that cinnamaldehyde is not a competitive inhibitor of AgrC-AIP binding. The results seem reasonable, because most of the reported inhibitors of the Agr QS system in S. aureus that can bind to AgrC are peptide mimetics of the native AIP (27).

AgrA contributes to Agr signaling in the physiology of L. monocytogenes as a response regulator; however, few reports on the determination of AgrA targets are available (14). Rieu et al. (15) reported that the transcription of the *agr* genes in an *agrA* in-frame deletion mutant was lower than that in parental strain EGD-e, indicating that the Agr system could be autoregulated by AgrA in L. monocytogenes. In our study, similar results were observed with the Δ*agrA* mutant of L. monocytogenes EGD-e. Furthermore, the absence of *agrA* significantly reduced the activity of *agr* promoter P_2_, suggesting that AgrA is a transcriptional activator of the P_2_ promoter. EMSA showed specific binding between AgrA and P_2_, which provided further evidence for positive regulation of the Agr system by AgrA. Our results suggest that cinnamaldehyde prevents AgrA from binding to the P_2_ promoter. After excluding the possibility of an interaction between cinnamaldehyde and P_2_, we proposed that AgrA was the target of cinnamaldehyde. Then, the binding of cinnamaldehyde to AgrA, determined by MST, supported our hypothesis.

Several small-molecule compounds, such as bumetanide (39) and F12 and F19 (40), have been reported to inhibit the Agr QS system in S. aureus by targeting AgrA. These inhibitors can interact with the DNA-binding domain of staphylococcal AgrA and block Agr from binding to promoter DNA (39, 40). AgrA is a member of the LytTR family response regulators whose DNA-binding domain is relatively uncommon among bacteria and is also called the LytTR domain (41–43). The AgrA of L. monocytogenes is characterized by a LytTR domain (residues 150 to 242) at the C-terminal end. In our study, cinnamaldehyde appeared to target the AgrA of L. monocytogenes and inhibit AgrA-P_2_ binding. Therefore, it is very likely that cinnamaldehyde can bind to the LytTR domain of AgrA. The predicted results from molecular docking demonstrated that seven amino acids of AgrA were involved in cinnamaldehyde binding. Except for His-29, the other six sites were located within the LytTR domain. The importance of conserved Asn-178 and Arg-179 in cinnamaldehyde-AgrA interactions was confirmed by alanine mutagenesis and subsequent MST. Furthermore, only Asn-178 was a key site for Agr-P_2_ binding. Cinnamaldehyde occupies Asn-178, leading to the decreased P_2_-binding ability of AgrA. Thus, our data indicate that cinnamaldehyde acts as a competitive inhibitor of AgrA-P_2_ binding. Notably, Asn-178 and Agr-179 are also highly conserved among strains of S. aureus, suggesting an interaction between cinnamaldehyde and AgrA in S. aureus.

Based on our results, a possible mechanism of cinnamaldehyde’s interference with the Agr system in L. monocytogenes was proposed. Cinnamaldehyde binds to the LytTR DNA-binding domain of AgrA, which prevents AgrA from binding to the P_2_ promoter and then inhibits the Agr system. However, several important questions about this mechanism remain answered. One is whether cinnamaldehyde affects AgrA phosphorylation. The AgrA of L. monocytogenes has an N-terminal phosphoacceptor receiver domain, in addition to the LytTR domain. The receiver domain transfers the phosphate group of phosphorylated AgrC to the Asp residue of AgrA and then catalyzes the autophosphorylation of AgrA (38, 44). We also noticed that only His-29 among all predicted sites was located in the N-terminal phosphoacceptor receiver domain of AgrA. Unfortunately, our data showed that this site was not associated with cinnamaldehyde-AgrA binding, suggesting that cinnamaldehyde did not affect AgrA activation upon phosphorylation. In the current study, the roles of all predicted sites in cinnamaldehyde-AgrA interactions were investigated; however, it cannot be excluded that cinnamaldehyde binds to other amino acid sites of AgrA.

The other question is whether cinnamaldehyde inhibits the biosynthesis of the signaling molecule AIP in L. monocytogenes. We posited that cinnamaldehyde, as an inhibitor of the Agr system, can affect AIP production. However, it is challenging to quantify L. monocytogenes AIP. At present, the structure of L. monocytogenes AIP is still inconclusive and controversial. Zetzmann et al. (45) reported a structure for L. monocytogenes AIP with the cyclic pentapeptide c-(Cys-Phe-Met-Phe-Val), while Todd et al. (46) proposed a hexapeptide with an alanine N-terminal extension from the same cyclic macrocycle, Ala-c-(Cys-Phe-Met-Phe-Val). The same strain (L. monocytogenes EGD-e) was used in these two studies. Therefore, the possibility that different structures of AIP resulted from strain differences was excluded. In addition, it is likely that L. monocytogenes cells produce a very small amount of AIP, because peptide signaling molecules are more metabolically costly, and their concentrations in bacterial culture are much lower than other types of signaling molecules (47). Further studies are needed to identify the structure of the L. monocytogenes AIP and to employ the appropriate method to evaluate the effects of cinnamaldehyde on AIP production.

L. monocytogenes can form biofilms on biological and abiotic surfaces during food processing, posing a threat to food safety and the food industry. Given that bacterial QS systems contribute to biofilm formation, interfering with the QS networks is considered to be a potential way to control food contamination by biofilms. Our results suggest that cinnamaldehyde can inhibit L. monocytogenes biofilm formation by interfering with the Agr QS system. Specifically, cinnamaldehyde binds to Asn-178 and Arg-179, which are located in the LytTR DNA-binding domain of AgrA. At the same time, Asn-178 plays an important role in the interaction between *agr* promoter P_2_ and AgrA. Therefore, the occupation of this site by cinnamaldehyde leads to decreased P_2_-AgrA binding. As a result, the transcription of the Agr system is suppressed, and biofilm formation is inhibited in L. monocytogenes.

## MATERIALS AND METHODS

### Bacterial strains and growth conditions.

Wild-type L. monocytogenes strain EGD-e was grown in brain heart infusion (BHI; Oxoid, Ltd., Basingstoke, Hampshire, England) broth at 37°C. The strains and plasmids used in this study are presented in Table S1 in the supplemental material.

### Determination of MIC.

The stock solution of cinnamaldehyde (>98% purity; Macklin Biochemical Co., Ltd., Shanghai, China) was prepared by dissolving cinnamaldehyde in ethanol. The agar dilution method was used to determine the MIC of cinnamaldehyde against L. monocytogenes EGD-e (48).

### Gene expression analyses.

The relative expression levels of the target genes were detected by qRT-PCR using the LightCycler 96 real-time PCR system (Roche, Basel, Switzerland). Primers for QS genes and motility-associated genes have been reported in our previous studies (49). L. monocytogenes EGD-e was grown in BHI medium with or without cinnamaldehyde (1/2 MIC). 16S rRNA was used as a reference gene, and the fold changes of the target genes were calculated using the cycle threshold (2^−ΔΔ^*^CT^*) method (50).

### Analysis of the *agr* promoter activity by β-galactosidase assays.

The *agr* promoter (P_2_)-*lacZ* fusion was constructed as described previously (51, 52). Briefly, the DNA fragment containing P_2_ was cloned into pPTPL, and the recombinant plasmid was electroporated into EGD-e. Transformants were selected by plating onto BHI agar plates with tetracycline (Sigma-Aldrich, St. Louis, MO, United States). A β-galactosidase activity assay based on the method of Miller was performed as described previously (53). The assays were performed in triplicate independently, and results were presented as the mean values in Miller units.

### Molecular docking.

The chemical structure of cinnamaldehyde was downloaded from the PubChem database (https://pubchem.ncbi.nlm.nih.gov/). The three-dimensional models of AgrA were generated by SwissModel (https://swissmodel.expasy.org/). The docking simulations were performed using AutoDock 1.5.6 tools (https://autodock.scripps.edu/), and PyMOL 2.3.0 (https://pymol.org/2/) was used to analyze the docking results.

### Expression of protein.

PCR primers were designed to obtain expression constructs for AgrC (the cytoplasmic domain [residues 207 to 431] is referred to as AgrC_Cyto_, and the entire protein is referred to as AgrC_FL_) and AgrA (the entire protein) (Table S2). The expression and purification of AgrC_FL_ were performed as described previously (54). The full-length open reading frame (ORF) of *agrC* was amplified and inserted into pET-28a and finally transformed into the expression host, E. coli strain C43(DE3). IPTG (isopropyl β-D-1-thiogalactopyranoside) was added to bacterial cultures with a final concentration of 0.1 mM to induce the expression of AgrC_FL_, and the cultures incubated for 24 h at 20°C. After centrifugation, the precipitate was resuspended in phosphate-buffered saline (PBS) containing 1 mM MgCl_2_, 1 mM protease inhibitor Pefabloc SC (Roche), 100 U/mL DNase, and 20 mM imidazole. Resuspended cells were lysed using ultrasonic waves and then centrifuged. Supernatants were centrifuged at 300,000 × *g* for 1 h at 4°C. The precipitates were resuspended with PBS containing 10× carboxymethyl cellulose (CMC) and 10 mM imidazole and then incubated on ice with shaking overnight. After centrifugation, supernatants containing the target protein AgrC_FL_ were collected. AgrC was purified using immobilized metal affinity chromatography (IMAC) and size exclusion chromatography (SEC). The purified AgrC_FL_ was analyzed via SDS-PAGE.

AgrC_Cyto_ and AgrA were expressed and purified according to the protocols described in our previous study (48). Briefly, the gene was inserted into pET-28a, and the recombinant vector was transformed into E. coli BL21(DE3) competent cells. The recombinant protein was expressed by adding IPTG and purified using BeyoGold His-tag purification resin (Beyotime Biotechnology Co., Shanghai, China).

### Detection of AgrC kinase activity.

AgrC_Cyto_ and AgrC_FL_ kinase activities were evaluated using a Kinase-Lumi chemiluminescence kinase activity detection kit (Beyotime) according to the manufacturer’s instructions. Briefly, the purified protein was incubated with the reaction buffer for 5 min at 37°C, and then ATP was added. After incubation for 10 min, the chemiluminescence of the samples was detected using a CLARIOstar multimode microplate reader (BMG Labtech, Offenburg, Germany). In the kinase assay, the amount of ATP remaining in the solution following a kinase reaction was measured, and luminescence was inversely related to kinase activity. To investigate the effect of cinnamaldehyde on AgrC_Cyto_ and AgrC_FL_ kinase activities, cinnamaldehyde at different concentrations was added to the mixture of protein and the reaction buffer, and then the chemiluminescence assay was performed as described above.

### MST.

The interaction between cinnamaldehyde and proteins was detected by MST using a Monolith NT.115 instrument (NanoTemper Technologies, Munich, Germany) according to the manufacturer’s instructions. Purified proteins were labeled using a protein-labeling kit (NanoTemper). Serial dilutions of cinnamaldehyde were prepared. The labeled protein was incubated with cinnamaldehyde at different concentrations. The samples were then filled into premium capillaries (NanoTemper), followed by measurements taken at 22°C. The interaction affinity and dissociation constant (*K_d_*) were analyzed using the MO.Control Analysis software (NanoTemper).

### Construction of the gene deletion mutant and the complementation mutant.

The temperature-sensitive shuttle vector pMAD was used to construct the gene deletion mutant of *agrA*, as described previously (55). In brief, an insert containing homologous arms up- and downstream from *agrA* was obtained by splicing by overlap extension PCR. The insert and pMAD were digested using BamHI and MluI. Then, the digested insert was ligated into pMAD using T4 ligase. The recombinant plasmid was electroporated into EGD-e, and transformants were selected on BHI plates with erythromycin (5 μg/mL; Sigma-Aldrich). Plasmid pERL3 was used for complementation experiments as described previously (56). The amplified product, included the coding sequences of *agrA*, was cloned into pERL3. The recombinant plasmid was first transformed into E. coli strain DH10β and was then electroporated into the Δ*agrA* mutant strain. Finally, transformants were selected on BHI plates with erythromycin.

### EMSA.

The DNA was incubated with purified recombinant AgrA in EMSA/gel-shift binding buffer (Beyotime Biotechnology Co., Shanghai, China) for 20 min at room temperature. To investigate the effect of cinnamaldehyde on the interaction between the P_2_ promoter and AgrA, cinnamaldehyde was added to the binding buffer. The DNA-protein complex was separated by 6% nondenaturing PAGE and visualized using ethidium bromide staining.

### Construction of a single mutation in AgrA by site-directed mutagenesis.

To identify the predicted active sites of AgrA, mutants were generated using the QuickMutation site-directed gene mutagenesis kit (Beyotime). The original vector, pET-28a-AgrA, was used as a template for PCR amplification. The primers are listed in Table S2. The nonmutated plasmid template was removed via digestion with DpnI for 1 h at 37°C. The expected mutants were confirmed by direct DNA sequencing. The mutant proteins were expressed and purified as described above.

### Effect of cinnamaldehyde on L. monocytogenes biofilm formation.

The microplate assay was performed to measure L. monocytogenes biofilms. Biofilms were incubated in 96-well polystyrene plates (Costar 3599; Corning, Inc., Kennebunk, ME, USA) and stained with crystal violet as described previously (57). To assess the influence of cinnamaldehyde on biofilm formation of L. monocytogenes, cinnamaldehyde was added to bacterial cultures with final concentrations of 1/8 MIC, 1/4 MIC, and 1/2 MIC. To determine the growth of planktonic cells, 100-μL amounts of cultures were centrifuged, and the pellets were resuspended in 1 mL of sterile saline. The bacterial cultures were 10-fold serially diluted, and 100-μL volumes were taken for colony counting.

### CLSM.

CLSM was used to observe biofilms as described previously (49). Fluorescent labeling of biofilms was performed using the Live/Dead BacLight bacterial viability kit (Molecular Probes, Eugene, OR, USA). After biofilm staining, image acquisition was performed using a Leica TCS-SP8 confocal laser scanning microscope (Leica Microsystems).

### Statistical analysis.

For statistical analysis of the results for gene transcription levels, β-galactosidase assays, biofilm biomass, and colony counting of biofilm bacteria, one-way analysis of variance (ANOVA) was carried out. The kinase activity assay results were analyzed using a two-way ANOVA model. Differences with *P* values of <0.05 were considered statistically significant.
