# Supplementary material for: Cinnamaldehyde Targets the LytTR DNA-Binding Domain of the Response Regulator AgrA to Attenuate Biofilm Formation of Listeria monocytogenes
Source: Microbiol Spectr. 2023 May 4;11(3):e00300-23. doi: 10.1128/spectrum.00300-23 (PMC10269664; doi:10.1128/spectrum.00300-23)
Supplement: Supplemental file 1 — Table S1. Download spectrum.00300-23-s0001.docx, DOCX file, 0.02 MB [file spectrum.00300-23-s0001.docx]

Table S1 Bacterial strains and plasmids used in this study.

| Strain or plasmid | Genotype^a^ | Reference or source |
| --- | --- | --- |
| Strain |  |  |
| *L. monocytogenes* |  |  |
| EGD-e | Wild-type strain; deposition number ATCC® BAA-679™ | A gift from Qin Luo |
| EGD-e pPTPL-P_2_ | EGD-e containing pPTPL-P_2_ | This study |
| Δ*agrA* | In-frame deletion of *agrA* | This study |
| CΔ*agrA* | Complemented strain of Δ*agrA* | This study |
| Δ*agrA* pERL3 | Δ*agrA* containing pERL3 | This study |
| *E. coli* |  |  |
| DH5α | Chemical competent strain | Biomed, Beijing, China |
| MC1000 | Cloning host for pPTPL | 50 |
| C43(DE3) | Expression host | Biomed, Beijing, China |
| BL21(DE3) | Expression host | Biomed, Beijing, China |
| DH10β | Chemical competent strain | Biomed, Beijing, China |
| Plasmid |  |  |
| pPTPL | Promoter probe vector, Tet^R^ | 50 |
| pPTPL-P_2_ | pPTPL containing the putative promoter region of the *agr* operon | This study |
| pET28a | Expression vector, Amp^R^ | Biomed, Beijing, China |
| pET28a-*agrC*_Cyto_ | pET28a containing the cytoplasmic domain of *agrC* | This study |
| pET28a-*agrC*_FL_ | pET28a containing the full length of *agrC* | This study |
| pET28a-*agrA* | pET28a containing the full length of *agrA* | This study |
| pMAD | Cloning shuttle integration vector plasmid, Amp^R^ and Ery^R^ | 54 |
| pMAD-Δ*agrA* | pMAD containing homologous arms up- and downstream of EGD-e *agrA* | This study |
| pERL3 | Plasmid capable of replication in *L. monocytogenes*, Ery^R^ | 55 |
| pERL3-*agrA* | pERL3 containing the upstream region and the coding sequence of *agrA* | This study |

^a^ TetR: resistance to tetracycline; Amp^R^: resistance to ampicillin; Ery^R^: resistance to erythromycin.
