# Supplementary material for: Cinnamaldehyde Targets the LytTR DNA-Binding Domain of the Response Regulator AgrA to Attenuate Biofilm Formation of Listeria monocytogenes
Source: Microbiol Spectr. 2023 May 4;11(3):e00300-23. doi: 10.1128/spectrum.00300-23 (PMC10269664; doi:10.1128/spectrum.00300-23)
Supplement: Supplemental file 2 — Table S2. Download spectrum.00300-23-s0002.docx, DOCX file, 0.02 MB [file spectrum.00300-23-s0002.docx]

Table 2 Primers used in this study.

| Application | Gene/protein | Primer name | Sequence (5'-3')^a^ |
| --- | --- | --- | --- |
| RT-qPCR | *luxS* | RTlmo1288-1 | AAGCACCTTTTGTGAGACTGG |
|  |  | RTlmo1288-2 | CCGTTAGTGTTGTAGCGATGA |
|  | *agrBD* | RTlmo0049-1 | GCTGGAAAGATGATGAAGAA |
|  |  | RTlmo0049-2 | TCTATCGGTCACTTTCGTATCT |
|  | *agrC* | RTlmo0050-1 | CTCTACAAAAGGAGAAGGTCGT |
|  |  | RTlmo0050-2 | TCTATCGGTCACTTTCGTATCT |
|  | *agrA* | RTlmo0051-1 | GCAAGCAGAAGAACGGATTT |
|  |  | RTlmo0051-2 | CTGTGGCACCGATAAAATGA |
|  | *flaA* | RTlmo0690-1 | GCTGGTATGAGTCGCCTTAG |
|  |  | RTlmo0690-2 | CATTTGCGGTGTTTGGTTT |
|  | *motA* | RTlmo0685-1 | TTTTACGGGATGTTTTGGAA |
|  |  | RTlmo0685-2 | TCGCTAAGTTTGTCTGGGTT |
|  | *motB* | RTlmo0686-1 | TTTGCTGACACTTTTACTTGC |
|  |  | RTlmo0686-2 | TCTTGTTCGTTTGCTTCTTTC |
|  | 16S rRNA | RT16S1 | GGGAGGCAGCAGTAGGGA |
|  |  | RT16S2 | CCGTCAAGGGACAAGCAG |
| Construction of P_2_-*lacZ* fusion | P_2_ | P-1 | *NNNNNN*AGATCTTAAAATGGAAAAGCCAACTG (*Bgl*II) |
|  |  | P-2 | *NNNNNN*TATAGACTAATTCACCTCCACTAATA (*Xba*I) |
| Expression of protein | AgrC_Cyto_ | AgrC-1 | *NNNNNN*GGATCCGCAACCAACGAACTTAAAG (*Bam*HI) |
|  |  | AgrC-2 | *NNNNNN*CTCGAGCTACATAATTTCTAATTCTTGAAT (*Xho*I) |
|  | AgrC_FL_ | AgrC-3 | *NNNNNN*GGATCCTTTAGTATTTTGATGGCAATTA (*Bam*HI) |
|  |  | AgrC-4 | *NNNNNN*CTCGAGCTACATAATTTCTAATTCTTGA (*Xho*I) |
|  | AgrA | AgrA-1 | *NNNNNN*GGATCCCTACCGGTTTTTATTTGTGA (*Bam*HI) |
|  |  | AgrA-2 | *NNNNNN*CTCGAGTAAACTCAAGCTTTTAATTAATTTC (*Xho*I) |
| Mutant strain construction | *agrA* | lmo0051-1 | *NNNNNN*GGATCCGCTCTTACTGTCTTAGCGTTCT (*Bam*HI) |
|  |  | lmo0051-2 | CCTGTGGCACCGATAAAATTCGCTGCATTCTGTTATCTTC |
|  |  | lmo0051-3 | GAAGATAACAGAATGCAGCGAATTTTATCGGTGCCACAGG |
|  |  | lmo0051-4 | *NNNNNN*ACGCGTAGTATTCCCATCGGCATTT (*Mlu*I) |
| Complementation | *agrA* | lmo0051-5 | *NNNNNN*GAGCTCGGATGAATTTATGCTACCGG (*Sac*I) |
|  |  | lmo0051-6 | *NNNNNN*GGATCCTAAACTCAAGCTTTTAATTAA *(BamHI)* |
| EMSA | P_2_ | P-3 | TAAAATGGAAAAGCCAACTG |
|  |  | P-4 | CTAATTCACCTCCACTAATA |
| Site-directed mutagenesis | AgrA_H29A_ | H29A-1 | TATATTATGGTTGAAGCTTTTGATATGAAGTTA |
|  |  | H29A-2 | TAACTTCATATCAAAAGCTTCAACCATAATATA |
|  | AgrA_D158A_ | D158A-1 | ATTCATGAACTGTTAGCTGATATCTTGTTTTTT |
|  |  | D158A-2 | AAAAAACAAGATATCAGCTAACAGTTCATGAAT |
|  | AgrA_D159A_ | D159A-1 | CATGAACTGTTAGACGCTATCTTGTTTTTTGAG |
|  |  | D159A-2 | CTCAAAAAACAAGATAGCGTCTAACAGTTCATG |
|  | AgrA_G176A_ | G176A-1 | AAAGTAATTTTACATGCTAAAAATCGCCAAGTG |
|  |  | G176A-2 | CACTTGGCGATTTTTAGCATGTAAAATTACTTT |
|  | AgrA_K177A_ | K177A-1 | GTAATTTTACATGGCGCTAATCGCCAAGTGGAA |
|  |  | K177A-2 | TTCCACTTGGCGATTAGCGCCATGTAAAATTAC |
|  | AgrA_N178A_ | N178A-1 | ATTTTACATGGCAAAGCTCGCCAAGTGGAATTT |
|  |  | N178A-2 | AAATTCCACTTGGCGAGCTTTGCCATGTAAAAT |
|  | AgrA_R179A_ | R179A-1 | TTACATGGCAAAAATGCTCAAGTGGAATTTTAT |
|  |  | R179A-2 | ATAAAATTCCACTTGAGCATTTTTGCCATGTAA |

^a^ Restriction sites are underlined. N, any of the bases.
